# Prediction of Functional Outcome in Ischemic Stroke Patients: An Observational Study on Egyptian Population

**DOI:** 10.7759/cureus.1392

**Published:** 2017-06-26

**Authors:** Mohamed Mahmoud Fouad, Sherien Mohamed Farag, Mohamed I Hegazy, Mohamed Abd Elalem Aziz

**Affiliations:** 1 Neurology, Ain Shams University; 2 Neurology, Kasr Al Aini Hospital; 3 Psychiatry, Amr Shahin Mental Hospital

**Keywords:** ischemic stroke, ischemic stroke predicators, functional outcomes, brain ischemia, three months outcome

## Abstract

Introduction

Determining the prognosis of ischemic stroke is important for neurologists and patients. The aim is to study the predictors of three months clinical outcome in ischemic stroke patients.

Materials and methods

A total of 397 patients were classified according to three months modified Rankin Scale score (mRS score) into two groups, favorable and unfavorable outcome. Favorable outcome was assumed if the score was zero or one, or unchanged if the score was ≥ 1 before the onset of the most recent event.

Results

The variables associated with unfavorable outcome were old age (*P *<0.001), presence of cardiac disease (*P *<0.001), low ejection fraction (*P*=0.008), low levels of total cholesterol and low-density lipoproteins (*P *<0.001), large artery atherosclerosis stroke (*P *<0.001), early confluent (*P*=0.005), high National Institute of Health stroke scale (NIHSS) score on admission (*P *<0.001), mRS score before admission (*P *<0.001), mRS score on discharge (*P *<0.001). Lacunar stroke was associated with favorable outcome (*P *<0.001). The regression analysis showed mRS score on discharge (*P *<0.001) and the presence of cardiac diseases (*P*=0.077) as independent predictors of unfavorable outcome.

Conclusion

High mRS score on discharge and presence of the cardiac disease independently could predict the unfavorable outcome and mRS score on discharge had a high sensitivity and negative predictive value in predicting the unfavorable outcome.

Abbreviations

MRS: score modified Rankin scale score, NIHSS: National Institutes of Health Stroke Scale, MRI: Magnetic resonance imaging, LDL: Low-density lipoprotein, MRA: Magnetic resonance of arteries.

## Introduction

The prognosis after ischemic stroke has always been a concern for the patients, their families, and the treating neurologists. This information is crucial in setting the management plan. Also, such information helps the researchers in the field of stroke to select the suitable patients in the clinical trials and to define the clinical endpoints [1]. A valid measure of outcome helps to reduce the time and cost of stroke trials [2]. The predictive models depend on the clinical features and investigatory tools such as brain imaging.

Multiple variables as age [3], severity of the clinical deficit assessed by National Institutes of Health Stroke Scale (NIHSS) [4 - 6] or modified Rankin scale (mRS) [5], cardiac disease [5, 7 - 9] non-lacunar stroke subtypes [10-11] moderate and severe leukoaraiosis [12] have been identified as potential predictors of poor clinical outcome in ischemic stroke. Informed consent statement was obtained for this study.

## Materials and methods

We followed the International Committee of Medical Journal Editors (ICMJE) STROBE checklist for observational studies during the preparation of the manuscript as we enrolled 397 patients with the diagnosis of acute ischemic stroke either as a first-ever or recurrent stroke. The patients were recruited consecutively from the stroke unit of Ain Shams University Specialized Hospital during the period from August 2015 till June 2016. The diagnosis was made based on the clinical features in combination with brain imaging. All patients were subjected to the stroke protocol and underwent magnetic resonance imaging (MRI) of the brain including diffusion weighted and gradient echo T2* weighted MRI scans. Leukoaraiosis (white matter hyperintensity) was graded using the scoring system [12] as absent white matter hyperintensities, punctuate white matter hyperintensities, early confluent white matter hyperintensities, and confluent white matter hyperintensities. Magnetic resonance of arteries (MRA) was visualized for the presence of intracranial arterial stenosis or occlusion. All of the patients were subjected to an electrocardiogram, transthoracic echocardiography, and carotid duplex for detection of stenosis of the extracranial carotid system. Further investigations were left to the discretion of the treating physician including transesophageal echocardiography.

Complete medical history was reviewed including age, gender and vascular risk factors such as hypertension (defined as history of use of antihypertensive medications or if systolic blood pressure was > 140 mmHg, diastolic blood pressure > 90 mmHg, or both during admission for four days at least), diabetes mellitus (defined as history of use of insulin or oral hypoglycemic agents or if blood glucose level was ≥ 126 mg/dl after an overnight fast or if it was ≥ 200 mg/dl after two hours from ingestion of 75 gms of oral glucose for at least two separate occasions).

Cardiac disease was considered if there was evidence of ischemic heart disease (such as acute myocardial infarction, angina, or coronary revascularization, low ejection fraction), atrial fibrillation, heart failure, and rheumatic heart disease. Past history of cerebrovascular events (ischemic stroke, transient ischemic attack, and intracerebral hemorrhage) was also recorded. Lipid profile was withdrawn for all patients.

Stroke severity was evaluated on admission using the NIHSS. Stroke subtypes were defined using the Trial of ORG 10172 in Acute Stroke Treatment (TOAST) criteria into one of five categories based on risk factors, as well as clinical and brain imaging features: large artery atherosclerosis, cardioembolism, small vessel occlusion (lacunar), undetermined etiology stroke or other aetiology [13-14].

The patients' functional status was assessed by the modified Rankin Scale (mRS) done on admission, on discharge from hospital and at three months follow-up. If the patient had a previous cerebrovascular stroke, the score of mRS was obtained before the onset of the most recent event. Favorable outcome was assumed if the mRS score was zero or one after three months from the onset of the stroke provided that mRS before the onset had been zero and whether it is the first-ever or recurrent stroke. If the patient had a history the of the previous stroke with mRS score ≥ 1 before the onset of the most recent event, favorable outcomes were assumed if the patient’s mRS score remains unchanged after three months.

Forty-two patients were excluded from the statistical analysis due to missing endpoint values, including patients with terminal illness and patients who missed three months follow-up mRS, and those who could not be tracked or refused to participate.

Statistical methods

The collected data were coded, tabulated, and statistically analyzed using IBM SPSS Statistics (Statistical Package for Social Sciences) software version 22.0 (IBM Corp., Armonk, New York, United States).

Descriptive statistics were done for quantitative data as minimum and maximum of the range, as well as mean ± standard deviation for quantitative parametric data, median and inter-quartile range for quantitative non-parametric data, while it was done for qualitative data as number and percentage.

Inferential analyses were done for quantitative variables using independent T-test in cases of two independent groups with parametric data and Mann-Whitney U test in cases of two independent groups with non-parametric data. In qualitative data, inferential analyses for independent variables were done using Chi-square test for differences between proportions and Fisher’s exact test for variables with small expected numbers. The multivariate logistic regression model was used to find out independent factors affecting unfavorable diagnosis. Receiver operating characteristic (ROC) curve was used to evaluate the performance of different tests and to differentiate between certain groups. The level of significance was taken at P value ≤ 0.05 as significant otherwise as non-significant.

## Results

Patients were divided into two groups; group I including 324 (81.6%) patients with favorable outcome and group II including 73 (18.4%) patients with unfavorable outcome.

1. Age and gender in study population

Group I included 203 males and 121 females, while group II included 44 males and 29 females with no significant difference when comparing both groups. The age of patients among group II (ranging from 40 to 89 years with mean age of 69.6±9.9 years) was significantly higher when compared to group I patients (ranging from 28 to 89 years with mean age of 61.2±10.3 years) (P <0.001) (Table 1).

**Table 1:** Age and gender in the study population

	Measures	All (n=397)	Group I (n=324)	Group II (n=73)	*P*
Age (years)	Mean ± SD	62.8±10.8	61.2±10.3	69.6±9.9	<0.001
	Range	28–89	28–89	40–89	
Gender	Female	150 (37.8%)	121 (37.3%)	29 (39.7%)	0.705
	Male	247 (62.2%)	203 (62.7%)	44 (60.3%)	

2. Vascular risk factor

*Hypertension: *Regarding the vascular risk factors (Table 2), hypertension was present in 274 patients (69% of study population). Although hypertension was more common among group II patients (75.3% of group population) compared to group I patients (67.6% of group population), this was statistically insignificant.

*Diabetes Mellitus: *Diabetes mellitus was detected in 211 patients (53.1% of study population), and its presence was nearly equal in both groups (53.1% of group I patients versus 53.4% of group II patients).

*Cardiac Diseases: *Cardiac diseases were present in 129 patients (32.5% of study population) and were significantly more common among group II patients (49.3% of group population) compared to group I patients (28.7 % of group population) (P< 0.001).

*History of Previous Ischemic Stroke: *A history of previous ischemic stroke was present in 112 patients (28.2% of study population), and this was significantly more common among group II patients (38.4% of group population) compared to group I patients (25.9% of group population) (P=0.033). There was no significant difference between both groups regarding a past history of transient ischemic attack (TIA) or intracerebral hemorrhage (ICH).

**Table 2:** Vascular risk factors in the study population

Variables	All (n=397)	Group I (n=324)	Group II (n=73)	*P*
Diabetes mellitus (DM)	211 (53.1%)	172 (53.1%)	39 (53.4%)	0.958
Hypertension (HTN)	274 (69.0%)	219 (67.6%)	55 (75.3%)	0.196
Cardiac diseases	129 (32.5%)	93 (28.7%)	36 (49.3%)	<0.001
Past history of ischemic stroke	112 (28.2%)	28 (38.4%)	93 (28.7%)	0.033
Past history of transient ischemic attack (TIA)	42 (10.6%)	35 (10.8%)	7 (9.6%)	0.761
Past history of intracerebral hemorrhage (ICH)	6 (1.5%)	3 (0.9%)	3 (4.1%)	0.079

3. Echocardiography findings

With regards to echocardiography findings, it was observed that the ejection fraction was significantly lower among group II patients (mean of 56.8±14.8%) compared to group I patients (mean of 61.9±8.8%) (P=0.008). Also, it was observed that the mean of left atrial diameter was higher among group II patients (a mean of 41.0±6.4) compared to group I patients (a mean of 38.8±6.4) (P=0.011).

4. Total cholesterol and low-density lipoprotein levels

The comparison of the laboratory data between both groups showed a statistically significant difference between the total cholesterol and low-density lipoprotein (LDL) levels, being lower among group II patients (mean of total cholesterol was 165.1±47.5 mg/dl and mean of LDL was 103.2±39.5 mg/dl), compared to group I patients (mean of total cholesterol of 189.5±49.1 mg/dl and mean of LDL was 122.3±41.4 mg/dl) (P <0.001) (Table 3).

**Table 3:** Lipid profile in the study population

Variables	Measures	All (n=397)	Group I (n=324)	Group II (N=73)	*P*
Total cholesterol (mg/dL)	Mean±SD	185.3±49.6	189.5±49.1	165.1±47.5	<0.001
	Range	60–363	66–363	60–334	
Triglycerides (mg/dL)	Mean±SD	146.2±88.2	149.5±87.6	130.2±89.8	0.105
	Range	19–653	40–653	19–518	
Low-density lipoprotein (mg/dL)	Mean±SD	118.9±41.7	122.3±41.4	103.2±39.5	<0.001
	Range	14–286	14–286	19–222	
High-density lipoprotein (mg/dL)	Mean±SD	37.7±11.9	37.7±12	37.9±11.6	0.933
	Range	7–153	7–153	11–68	

5. Presence of microbleeds and leukoaraiosis

Leukoaraiosis was detected among 197 patients (60.8%) of group I population and 52 patients (71.2%) of group II population. It was found that early confluent and confluent leukoaraiosis were significantly more common among group II patients compared to group I patients. Early confluent leukoaraiosis was detected in 34.2% of group II patients compared to 18.5% of group I patients (P=0.005), and confluent leukoaraiosis was detected in 26% of group II patients compared to 15.4% of group I patients (P=0.018). There was no significant difference between both groups regarding the presence of microbleeds (Table 4).

**Table 4:** Microbleeds and leukoaraiosis in the study population

	Grade	All (n=397)	Group I (n=324)	Group II (n=73)	*P*	OR (95% CI)
Micro- Bleeds	Absent	307 (77.3%)	253 (78.1%)	54 (74.0%)	Reference	
	Mild	48 (12.1%)	39 (12%)	9 (12.3%)	0.845	1.08 (0.50–2.36)
	Moderate	35 (8.8%)	27 (8.3%)	8 (11%)	0.443	1.39 (0.60–3.22)
	Severe	7 (1.8%)	5 (1.5%)	2 (2.7%)	0.453	1.87 (0.35–9.16)
	Present	90 (22.7%)	71 (21.9%)	19 (26%)	0.448	1.25 (0.70–2.25)
Leuko- araiosis	Absent	148 (37.3%)	127 (39.2%)	21 (28.8%)	Reference	
	Punctuate	95 (23.9%)	87 (26.9%)	8 (11%)	0.176	0.56 (0.24–1.31)
	Early confluent	85 (21.4%)	60 (18.5%)	25 (34.2%)	0.005	2.50 (1.31–4.86)
	Confluent	69 (17.4%)	50 (15.4%)	19 (26%)	0.018	2.30 (1.14–4.64)
	Present	249 (62.7%)	197 (60.8%)	52 (71.2%)	0.096	1.60 (0.92–2.78)

6. Stroke subtypes

Regarding the subtypes of stroke, it was noticed that large artery atherosclerosis stroke had been more common among group II patients compared to group I patients with a highly significant statistical difference (P ˂0.001), unlike the lacunar stroke which was more common in group I compared to group II (P ˂0.001). There was no significant difference between both groups regarding cardioembolic stroke (P=0.697) and stroke of undetermined etiology (P=0.167) (Table 5).

**Table 5:** Subtypes of stroke among the study population

Type of stroke	All (n=397)	Group I (n=324)	Group II (n=73)	P
Large artery atherosclerosis	169 (42.6%)	119 (36.7%)	50 (68.5%)	<0.001
Cardioembolic	11 (2.8%)	10 (3.1%)	1 (1.4%)	0.697
Small vessel disease	209 (52.6%)	190 (58.6%)	19 (26%)	<0.001
Stroke of undetermined etiology	8 (2.0%)	5 (1.5%)	3 (4.1%)	0.167

7. National Institute of Health stroke scale (NIHSS) and modified Rankin Scale (mRS) scores

The NIHSS score done on admission among the group I patients ranged from one to 21 with median interquartile range (IQR) of seven, while among group II patients ranged from three to 23 with median (IQR) of 12. Comparing both groups showed that group II patients tended to have significantly higher NIHSS scores (P ˂0.001). Group II patients had significantly higher mRS score before admission with median (IQR) of five compared to group I patients who had median (IQR) of four (P ˂0.001) and higher mRS score on discharge with median (IQR) of five compared to group I patients who had median (IQR) of two (P ˂0.001) (Table 6).

**Table 6:** National Institutes of Health Stroke Scale (NIHSS) score among the study population

Variables	Measures	All (n=397)	Group I (n=324)	Group II (n=73)	*P*
NIHSS score on admission	Median (IQR)	8 (5–11)	7.0 (4–10)	12 (8–15.5)	<0.001
	Range	1–23	1–21	3–23	
mRS score before admission	Median (IQR)	4 (2–4)	4 (2–4)	5 (4–5)	<0.001
	Range	0–5	1–5	0–5	
mRS score on discharge	Median (IQR)	3 (2–4)	2 (2–4)	5 (3–6)	<0.001
	Range	0–6	0–5	1–6	

After adjustment for all variables and at regression analysis, mRS score on discharge (P <0.001, 95% CI=1.878 [1.533‒2.301]) and the presence of cardiac disease (P=0.063, 95% CI=1.657 [0.972‒2.825]) were the most likely factors related to unfavorable outcome among all the study population. When regression analysis was done among the population with no past history of ischemic stroke, mRS score on discharge (P=0.011, 95% CI=1.574 [1.110‒2.234]) and cardiac disease (P=0.077, 95% CI=2.242 [0.918‒5.479]) were retained as predictors of unfavorable outcome and when done among the population with past history of ischemic stroke, mRS score on discharge was again retained as predictor of unfavorable outcome (P <0.001, 95% CI=2.032 [1.578‒2.618]) (Table 6).

**Figure 1:**
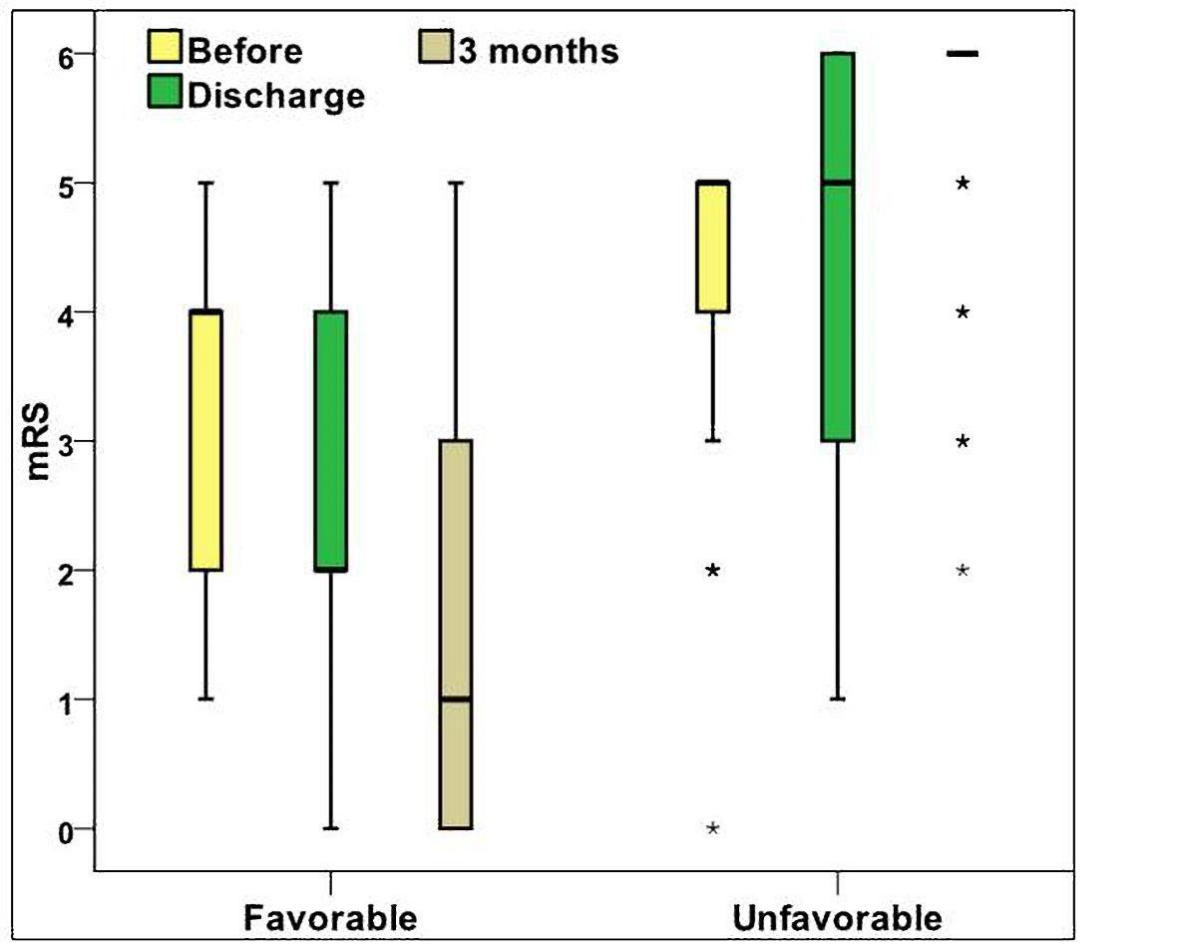
Modified Rankin Scale (mRS) scores among the study groups

The diagnostic performance of mRS score on discharge in prediction of unfavorable outcome revealed that mRS score on discharge had a high sensitivity (95% CI=86.3 [76.6‒92.8]) and negative predictive value as (95% CI=94.2 [90.1‒96.9]) for prediction of unfavorable outcome (Figure 1).

## Discussion

This retrospective observational study showed a comparison between the data of two groups of patients, a group of patients with favorable outcome versus another group with unfavorable outcome. The mRS, being one of the most widely used measures of assessment of the outcome of stroke, was used to assess three months outcome. Favorable outcome represented 81.6% of the study population and unfavorable outcome represented 18.4% of study population.

In agreement with the literature, old age was significantly correlated with unfavorable outcome (P <0.001). Advancing age has a major negative impact on stroke morbidity, mortality and long-term outcome [6, 15]. People older than 65 years of age had increased risk of mortality after two months from onset of stroke and admission to a skilled nursing facility [16-17].

Regarding the vascular risk factors, the presence of cardiac disease was significantly associated with unfavorable outcome (P <0.001). Nearly 50% of group II patients had cardiac disease. The presence of cardiac disease was shown to be a predictor of unfavorable outcome after regression analysis (P=0.063). This goes with the observation that group II patients tend to have lower ejection fraction when compared to group I patients (P=0.008). However, low ejection fraction alone was not retained as a predictor of unfavorable outcome after regression analysis, similar to two other studies [6, 18-19].

The relation between the different cardiac disease and poor outcome was illustrated in many studies; congestive heart failure [14, 18], non-valvular atrial fibrillation [7, 18], coronary artery disease was associated with poor outcome and risk factors for dependency [18]. This raises the importance that patients with cardiac diseases shall receive an optimum care to prevent the occurrence of ischemic stroke and shall be carefully monitored at the hospital if the ischemic stroke occurs in order to decrease the mortality.

The patients who had a past history of ischemic stroke tended to have the unfavorable outcome (P=0.033). This was corroborated by many studies [4, 20] and raises the issue that patients with past history of stroke shall have careful management and monitoring in order to prevent recurrence and avoid worse prognosis.

An interesting observation was that patients with unfavorable outcome tended to have lower levels of total cholesterol and LDL (P <0.001). The association between cholesterol levels and stroke outcome is controversial. Some studies supported this study finding; in one study, low cholesterol levels increases long-term dependency and recurrence rate but do not increase mortality rates [21]. Moreover, two other studies reported that low cholesterol levels were associated with increased short and long-term mortality rates, as well as increased risk of severe stroke suggesting that the epidemiology should be reversed in the association between cholesterol and outcome in patients with ischemic stroke [21-22]. The mechanism explaining the association between cholesterol levels and poor stroke outcomes is unknown and needs to be validated.

A highly significant association between the severity of stroke, assessed by NIHSS score on admission and an unfavorable outcome was detected (P ˂0.001). Several studies have demonstrated that the NIHSS score was a good predictor of stroke outcome [4, 19] and that a remarkable neurologic impairment assessed by NIHSS score was associated with less favorable outcome [15]. In one study, the NIHSS score at one week was highly predictive of three months outcome; even more the addition of the infarct volume did not improve the accuracy of the predictive model [23].

High scores of mRS before admission (P ˂0.001) and mRS on discharge (P ˂0.001) had been more common among group II patients. Dependency before stroke onset is commonly associated with poor outcome [17]. The mRS score on discharge was retained as an important predictor of unfavorable outcome after regression analysis (P <0.001). One study reported that mRS score at seven to 10 days from onset of ischemic stroke was independently correlated to mRS score at three months [4]. Also, the mRS score on discharge was proven to have a high sensitivity and negative predictive value in predicting unfavorable outcomes.

Regarding MRI findings, it was noticed that the presence of early confluent and confluent leukoaraiosis was significantly associated with unfavorable outcome (P=0.005 and P=0.018 respectively). In previous studies, moderate and severe leukoaraiosis were related to poor outcome [11] and associated with patient mortality .

In this study, an evident relationship between lacunar stroke and favorable outcome was noticed (P ˂0.001) and this was adopted in many studies where lacunar infarcts were associated with short-term good prognosis with decreased mortality and dependency [24]. This may be conflicting when related to the above-mentioned observation that leukoaraiosis had been related to unfavorable outcome as both lacunar stroke and leukoaraiosis are reflecting the presence of small vessel diseases, but it seems that lacunar stroke is the early stage of small vessel disease and leukoaraiosis is the late one. Lacunar infarcts are related to a worse long term prognosis with increased risk of death, stroke recurrence and dementia. For this reason, lacunar infarction should be regarded as potentially serious rather than a relatively benign disorder and, therefore, lacunar stroke patients require monitoring [25].

Many studies reported that patients with strokes of cardio-embolic or large artery etiology tend to have worse prognosis compared with other ischemic stroke subtypes [26-28], and this study reported a significant association between large artery atherosclerosis stroke and unfavorable outcome (P ˂0.001).

The limitations of our study were the retrospective design, limited sample size and the non-use of thromblytic therapy which is expensive and are not commonly available at our stroke centers. 

The strengths of this study include the recruitment of consecutively admitted patients with acute ischemic stroke, thus allowing homogenous stroke population and avoiding selection bias. Also, the clinical criteria and the investigation modalities used in determination of the outcome can be easily performed and are indeed included in the routine stroke management protocol. This issue has been our major concern as we seek to provide the predictors of outcome that are cost-beneficial and ones that can be applied in the daily clinical practice at our community.

## Conclusions

The modified Rankin Scale (mRS) score on discharge and the presence of cardiac disease independently predicted the unfavorable outcome of ischemic stroke at three months follow-up. The mRS score on discharge had a high sensitivity and negative predictive value in predicting the unfavorable outcome.
